# Long noncoding RNA expression profile and association with SLEDAI score in monocyte-derived dendritic cells from patients with systematic lupus erythematosus

**DOI:** 10.1186/s13075-018-1640-x

**Published:** 2018-07-11

**Authors:** Yilun Wang, Shuang Chen, Sunyi Chen, Juan Du, Jinran Lin, Haihong Qin, Jie Wang, Jun Liang, Jinhua Xu

**Affiliations:** 1 Department of Dermatology, Huashan Hospital, Fudan University, 12 Wulumuqi Zhong Road, Shanghai, 200040 People’s Republic of China; 2 Department of Human Anatomy and Histoembryology, School of Basic Medical Science, Fudan University, Shanghai, People’s Republic of China

**Keywords:** Systematic lupus erythematosus, Monocyte-derived dendritic cells, Long noncoding RNA, Expression profile

## Abstract

**Background:**

Monocyte-derived dendritic cells (moDCs) play important roles in the pathogenesis of systemic lupus erythematosus (SLE). Aberrant expression of long noncoding RNAs (lncRNAs) could affect the function of moDCs. The aim of this study was to explore the lncRNA expression profile in moDCs of SLE patients to provide new insights into SLE.

**Methods:**

LncRNA and mRNA microarrays were performed to identify differentially expressed lncRNAs and mRNAs in moDCs of SLE patients compared with normal controls. Bioinformatics analysis was also performed. Quantitative polymerase chain reaction (qPCR) was used to validate the results, and correlation analysis was used to analyze the relationship between these aberrantly expressed lncRNAs and SLE disease activity index (SLEDAI) scores.

**Results:**

According to the gene expression profiles, 163 lncRNAs were differentially expressed between SLE and normal controls, including 118 that were upregulated and 45 that were downregulated. A total of 137 mRNAs were differentially expressed in moDCs of patients with SLE, including 83 that were upregulated and 54 that were downregulated. Furthermore, qPCR data showed that lncRNA ENST00000604411.1 (18.23-fold, *P* < 0.001) and ENST00000501122.2 (1.96-fold, *P* < 0.001) were upregulated and the other two lncRNAs, lnc-HSFY2–3:3 (0.42-fold, *P* < 0.001) and lnc-SERPINB9–1:2 (0.50-fold, *P* = 0.040), were downregulated in moDCs of SLE patients. The expression levels of ENST00000604411.1 (*r* = 0.593, *P* = 0.020) and ENST00000501122.2 (*r* = 0.539, *P* = 0.038) were positively correlated with the SLEDAI score, respectively.

**Conclusions:**

The results indicate that the abnormal expression of lncRNAs in moDCs may be involved in the pathological processes of SLE. The expression level of ENST00000604411.1 and ENST00000501122.2 may have potential value for the assessment of disease activity in SLE.

**Electronic supplementary material:**

The online version of this article (10.1186/s13075-018-1640-x) contains supplementary material, which is available to authorized users.

## Background

Systematic lupus erythematosus (SLE) is an autoimmune disease that may damage multiple organs by autoantibodies and immune complexes. The precise etiology of SLE is still unclear and it might involve the regulation of genes, environments, and immune imbalance. Experimental evidence suggests that the pathogenesis of SLE is related to the failure of T- and B-cell suppression mediated by defects in cell signaling, immune tolerance, and apoptotic mechanisms promoting autoimmunity [1].

Although T and B cells have been widely studied in SLE, the upper stream cells which could present autoantigens to them have only been emphasized more recently. Autoantigens are released mainly from secondary necrotic cells because of a defective clearance of apoptotic cells in patients with SLE [2]. Dendritic cells (DCs) are the most efficient antigen-presenting cells (APCs) in the human body. Recent research has associated lupus development with changes in the DC compartment, including altered DC subset frequency, localization, phenotype, and functional defects [3]. The dysfunction of DCs is related to the overreaction of T cells and B cells in SLE patients [4], including presenting autoantigens to autoreactive T cells, overproduction of proinflammatory cytokines and chemokines, suppression of Tregs, and promoting B cells to secret autoantibodies [5–8], which results in the loss of self-tolerance and production of autoantibodies.

Recent advances suggest that long noncoding RNAs (lncRNAs) regulate gene expression at the pretranscriptional, transcriptional, and post-transcriptional levels [9]. LncRNAs mediate their molecular functions through a multitude of mechanisms [10]. An lncRNA could interact with proteins in the cytoplasm as a guide, scaffold, or decoy molecule [10]. LncRNAs could also promote or repress the translation of mRNAs in the cytoplasm. For example, the antisense lncRNA BACE1-AS rapidly and reversibly upregulates BACE1 levels in response to a variety of stresses. Since BACE1 and BACE1-AS form an RNA duplex, the duplex may act to alter the secondary or tertiary structure of BACE1 and thereby increase its stability [11]. In the nucleus, lncRNAs can act in *cis* to control local allele-specific functions or in *trans* at one or more genomic loci to regulate gene expression.

LncRNAs are important in regulating the differentiation and function of DCs. Lnc-DC, which is exclusively expressed in human conventional DCs, can affect cellular differentiation (monocytes into dendritic cells) and reduce the capacity of DCs to stimulate T-cell activation by activating the transcription factor STAT3 [12]. The expression of the lncRNA HOTAIRM1 was downregulated when monocytes differentiated into DCs, and silencing of HOTAIRM1 caused changes in the expression of several monocyte differentiation markers such as CD14 and B7H2 [13]. As a result, lncRNAs are able to cause clinical diseases involving the dysfunction of DCs. However, it is largely unknown whether lncRNAs participate in the pathogenesis of SLE by regulating the function of DCs; this is therefore the focus of this study.

## Methods

### Subjects

Fifteen female SLE patients were recruited from the inpatient service in Huashan Hospital, Fudan University. The diagnostic criteria were in accordance with the 1997 American College Rheumatology revised criteria for the classification of SLE. Relevant clinical and laboratory information regarding the patients is shown in Table 1. Fifteen age-matched female healthy controls were also recruited. The study was approved by the Independent Ethics Committee of Huashan Hospital and written informed consent was obtained from all subjects. All the experiments were carried out in accordance with relevant guidelines and regulations of Huashan Hospital.

**Table 1:** Clinical and laboratory characteristics of the patients with SLE in the study

Characteristic	SLE (*n* = 15)
Sex, male/female (*n*)	0/15 (15)
Age (years), median (range)	33 (17–57)
Duration (months), median (range)	3 (1–60)
SLEDAI score, median (range)	15 (10–30)
ANA > 1:320, yes/no (*n*)	15/0 (15)
Anti-dsDNA (IU/ml), median (range)	391.9 (25.9–800)
Hypocomplementemia, yes/no (*n*)	12/3 (15)
Organ involvement, yes/no (*n*)	6/9 (15)
Steroids, yes/no (*n*)	9/6 (15)
Immunosuppressive drugs, yes/no (*n*)	15/0 (15)

*ANA* antinuclear antibody, *SLE* systemic lupus erythematosus, *SLEDAI* systemic lupus erythematosus disease activity index

### Cell culture

In-vitro differentiation of human monocytes into DCs was performed as described previously [8]. Briefly, peripheral blood mononuclear cells (PBMCs) were isolated and CD14^+^ monocytes were sorted by positive selection using magnetic beads (Miltenyi Biotec, Germany). The cells were then cultured for 5–7 days in RPMI 1640 supplemented with 1000 U/ml granulocyte/macrophage colony-stimulating factor (GM-CSF) and 1000 U/ml interleukin (IL)-4 (PeproTech, Rocky Hill). For monocyte-derived dendritic cell (moDC) maturation, 1 μg/ml lipopolysaccharide (LPS; *Escherichia coli* type 055:B6; Sigma) was added to the medium at day 6. The following antigens were used to identify the phenotype of moDCs: CD11c, HLA-DR, CD40, CD86, CD83, and low expression of CD14. The antibodies were all bought from eBioscience (San Diego).

### LncRNA and mRNA microarray

LncRNA expression profiling was determined by Human 4*180 K lncRNA arrays manufactured by Agilent Technologies (Santa Clara, CA), including 63,431 lncRNA probes and 39,887 mRNA probes. Each transcript was represented using 1–5 probes to improve statistical confidence. The lncRNA and mRNA expression data have been deposited into the Gene Expression Omnibus (GEO) under accession number GSE89240.

### Gene function analysis

The Database for Annotation, Visualization and Integrated Discovery (DAVID; https://david.ncifcrf.gov) was utilized to identify the molecular function represented in the gene profile. Furthermore, the Kyoto Encyclopedia of Genes and Genomes (KEGG) database (https://www.genome.jp/kegg/) was also used to analyze the potential functions of these target genes in the pathways. The cut-off criterion for false discovery rate (FDR) was set at less than 0.05.

### Coexpression network construction

The lncRNA-mRNA coexpression network was constructed based on the correlation between the differentially expressed lncRNAs and mRNAs using Cytoscape. Pearson correlation analysis was used to evaluate the significance of the correlation between the expression levels between each pair of genes. Pearson correlation coefficients were selected for inclusion in the network when they were above 0.95.

### *Cis* and *trans* target gene analysis of lncRNAs

The gene location for different lncRNAs on the chromosome was determined. Subsequently, the common lncRNA coexpression genes were intersected to identify the genes 10 kbp upstream or downstream of the lncRNAs as potential ‘*cis*’ genes. For the *trans* analyses, we predicted the *trans*-associated genes of the differentially expressed lncRNAs with RNAplex v0.2. The RNAplex parameters were set as e ≤ −30 in the current study to identify the *trans*-associated genes, and genes that were found to be located on the same chromosome as the lncRNA were excluded.

### Quantitative real-time polymerase chain reaction (qRT-PCR)

Total RNA was reverse transcribed using a PrimeScript RT reagent Kit with gDNA Eraser (Perfect Real Time; TaKaRa, Dalian, China) in accordance with the manufacturer’s instructions. The expressions of selected lncRNAs were analyzed using qRT-PCR with an ABI Power SYBR Green PCR Master Mix (ABI, USA). Glyceraldehyde 3-phosphate dehydrogenase (GAPDH) mRNA was used as an internal control. The primers are listed in Table 2. For quantitative results, expression of each lncRNA was represented as a fold change using the 2^–ΔΔCt^ method and then statistically analyzed.

**Table 2:** The sequences of quantitative polymerase chain reaction primers

Primer name	Sequence (5’ to 3’)
gapdh(Hs) forward	TGACTTCAACAGCGACACCCA
gapdh(Hs) reverse	CACCCTGTTGCTGTAGCCAAA
ENST00000604411.1 forward	AGCCCCACTTCACATTAGACC
ENST00000604411.1 reverse	TGATGTTGCAGTCCTGTGAGG
ENST00000501122.2 forward	TTCTGCTTTCTGCCCATGTA
ENST00000501122.2 reverse	GTGGCAGTGACAACCTCTCA
ENST00000568394.1 forward	TCTCTCCCCAGTGACAGTACA
ENST00000568394.1 reverse	GCGATCTGTAGTCCAGGTGT
lnc-HSFY2–3:3 forward	TGATTGGAAGATGGAAGTGGA
lnc-HSFY2–3:3 reverse	GCTCCACCTGAAACTCATTTG
lnc-SERPINB9–1:2 forward	GGGGAAAATAAAAGGGATGGT
lnc-SERPINB9–1:2 reverse	TCTCTCTTAGGGAAGGGCATT

### Statistics

Continuous variables are expressed as means (SD) and categorical variables as frequencies (%). The Student *t* test or one-way analysis of variance was used to compare continuous variables. All *P* values were estimated in a two-tailed fashion. Differences were considered to be statistically significant at *P* < 0.05. Data were analyzed using SPSS 13.0 (SPSS Inc., IL, USA). The relationships between the expression levels of lncRNAs and systemic lupus erythematosus disease activity index (SLEDAI) were analyzed by Pearson’s correlation coefficient.

## Results

### Differential expression profile of lncRNAs in moDCs of SLE

**Fig. 1:**
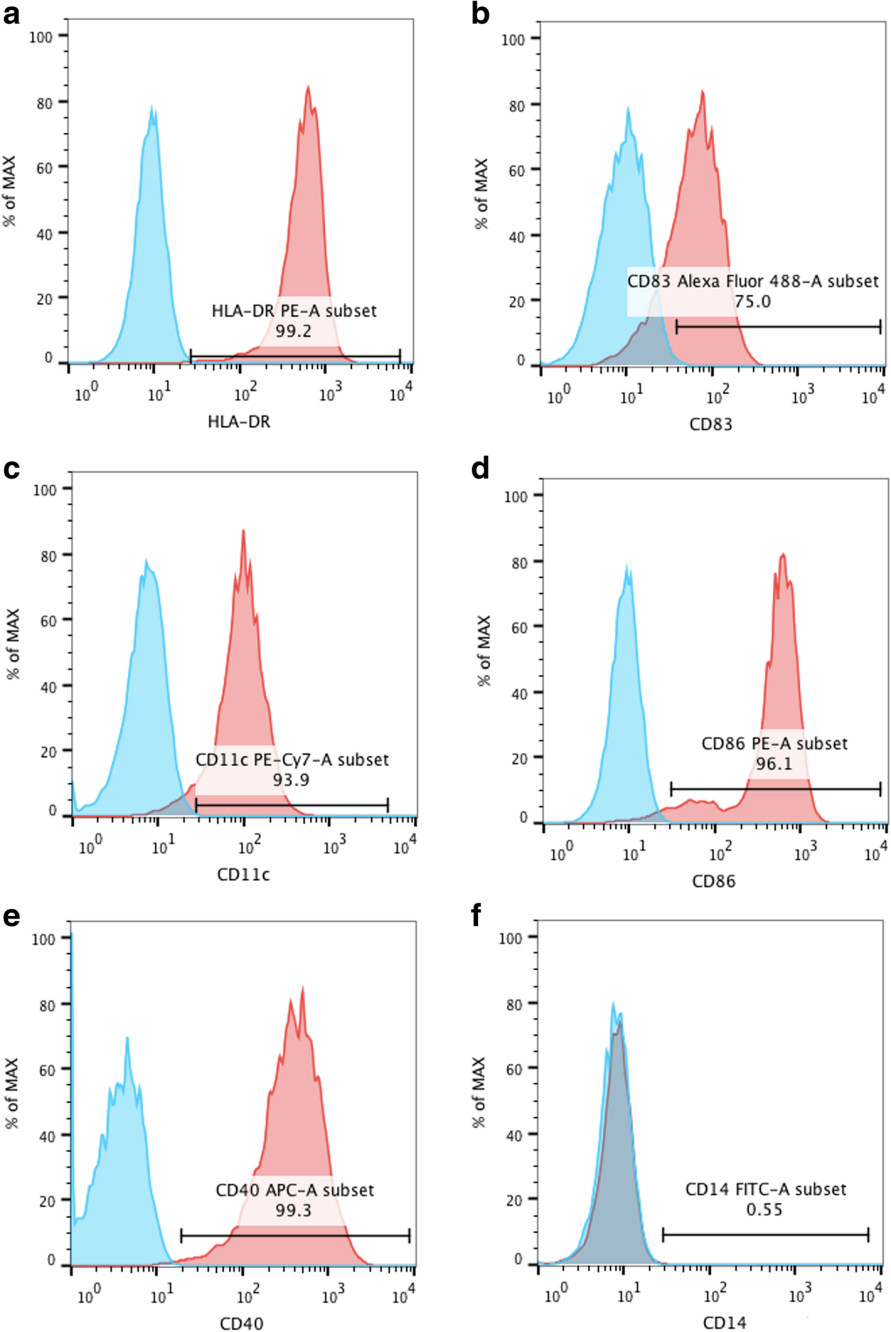
moDCs were identified using flow cytometry. The phenotype of the cells displayed high expression of **a** HLA-DR, **b** CD83, **c** CD11c, **d** CD86, and **e** CD40, and **f** low expression of CD14

**Fig. 2:**
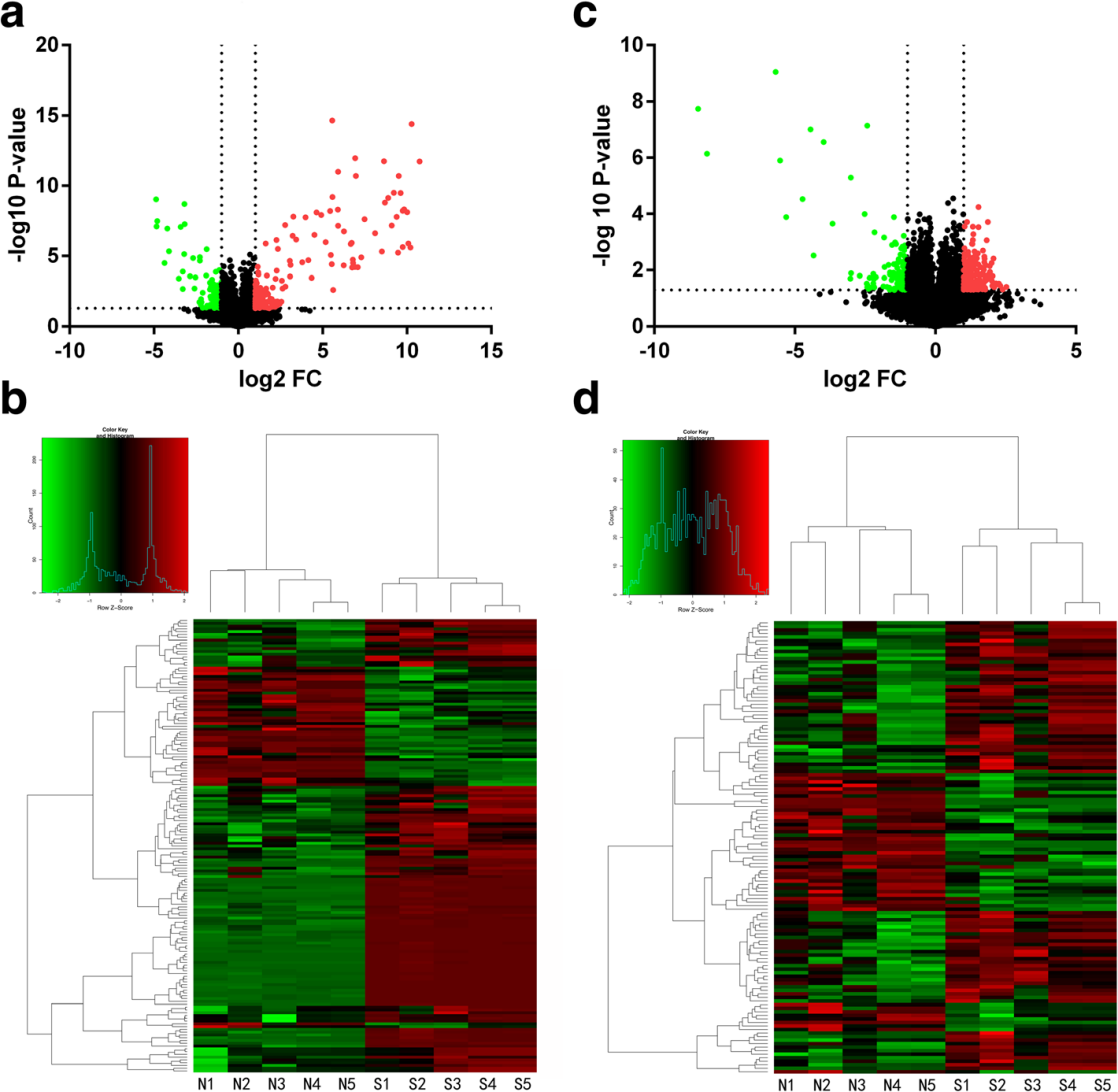
Volcano plots and hierarchical clusters of differentially expressed lncRNAs and mRNAs. **a** LncRNA volcano plots of SLE patients versus normal controls. **b** A total of 163 lncRNAs were differentially expressed in moDCs of patients with SLE (*n* = 5) compared with normal controls (*n* = 5), including 118 upregulated and 45 downregulated lncRNA (fold change (FC) ≥ 2.0, *P* < 0.05). **c** mRNA volcano plots of SLE patients versus normal controls. **d** A total of 137 mRNAs were differentially expressed in moDCs of patients with SLE (*n* = 5) compared with normal controls (*n* = 5), including 83 upregulated and 54 downregulated mRNAs (fold change ≥ 2.0, *P* < 0.05). In **a** and **c**, each point represents a different transcript. Red points represent genes that were significantly upregulated and green points represent genes that were significantly downregulated. In **b** and **d**, relatively high expression is indicated by red shading and relatively low expression is indicated by green shading. N1–5 represents normal controls and S1–5 represents the patients with SLE

Dendritic cells were differentiated from CD14^+^ monocytes. MoDCs were identified using flow cytometry. The phenotype of moDCs displayed high expression of HLA-DR, CD83, CD11c, CD86, and CD40, and low expression of CD14 (Fig. 1). To profile differentially expressed lncRNAs in moDCs of patients with SLE, we performed a genome-wide analysis of lncRNA expression in moDCs between SLE patients and normal controls. Volcano plot analysis showed that 163 (0.22%) of the lncRNAs were significantly differentially expressed in moDCs of patients with SLE (Fig. 2a), including 118 upregulated and 45 downregulated lncRNAs (fold change ≥ 2.0, *P* < 0.05) (Fig. 2b). We categorized these differentially expressed lncRNAs into six classes: intergenic, intronic sense, intronic antisense, bidirectional, exonic sense, and exonic antisense. We observed 150 intergenic, 2 intronic sense, 1 intronic antisense, 5 bidirectional, 4 exonic sense, and 1 exonic antisense lncRNA (Additional file 1: Table S1).

### Differential expression profile of mRNAs in moDCs of SLE

**Fig. 3:**
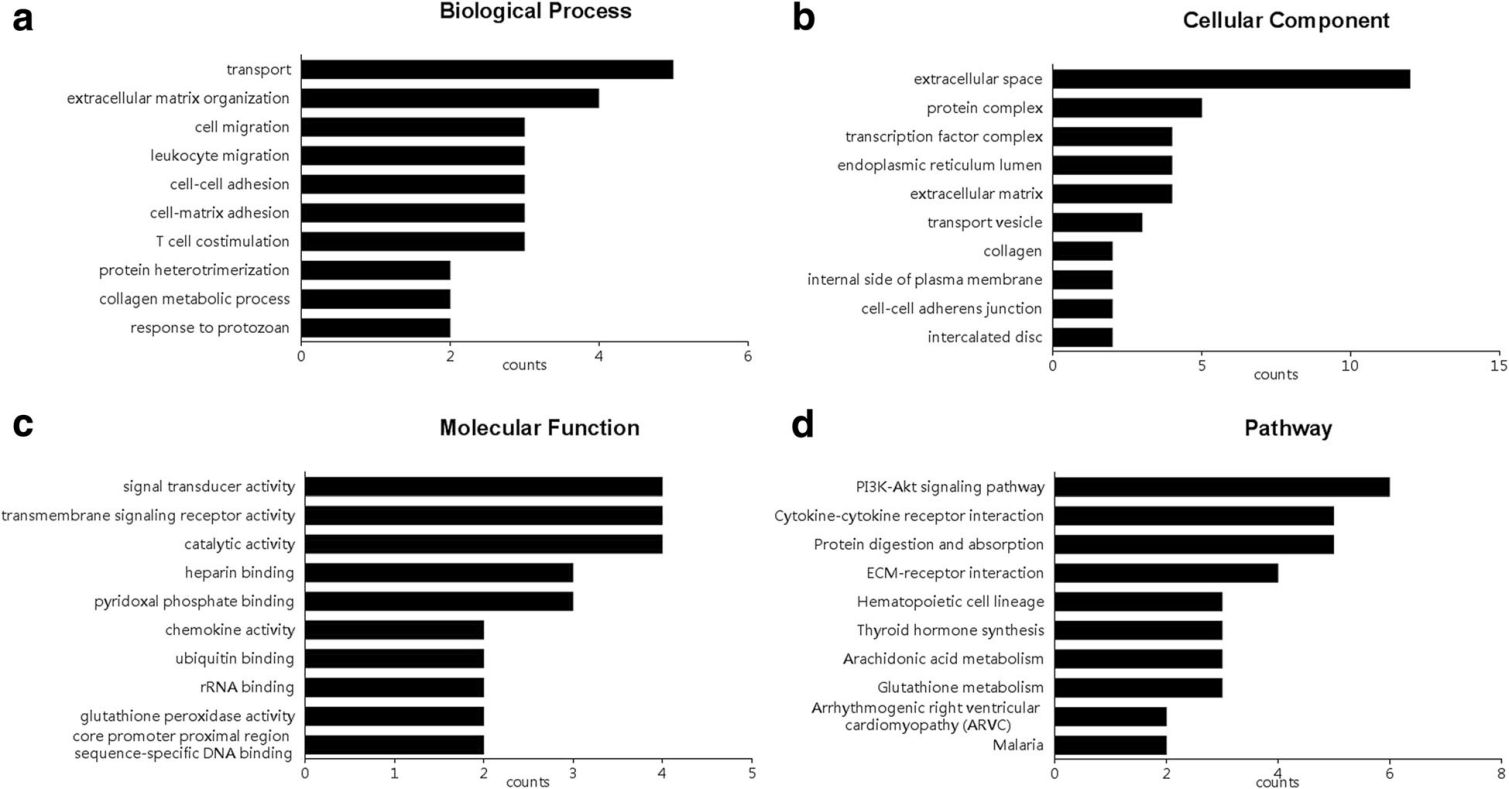
Gene ontology (GO) and pathway enrichment analysis for differentially regulated mRNAs. **a** GO analysis of mRNAs according to biological process. **b** GO analysis of mRNAs according to cellular component. **c** GO analysis of mRNAs according to molecular function. **d** Pathway analysis of differentially expressed mRNAs in moDCs of SLE patients. ECM extracellular matrix

A total of 137 (0.34%) mRNAs were differentially expressed in moDCs of patients with SLE compared with normal controls (fold change ≥ 2.0, *P* < 0.05) (Fig. 2c). Among them, 83 mRNAs were upregulated and 54 mRNAs were downregulated (Fig. 2d, Additional file 2: Table S2). Gene ontology (GO) analysis was performed to classify these differentially expressed mRNAs into three domains: biological process, molecular function, and cellular component. In the biological process domain, the GO terms for the differentially expressed mRNAs included cell migration, cell-cell adhesion, and T-cell costimulation, etc. (Fig. 3a). In the cellular component domain, the top five GO terms were extracellular space, protein complex, transcription factor complex, endoplasmic reticulum lumen, and extracellular matrix (Fig. 3b). In the molecular function domain, the GO terms contained signal transducer activity, chemokine activity, and ubiquitin binding (Fig. 3c). KEGG pathway analysis was also conducted to identify the key signaling pathways and the relationships among the differentially expressed mRNAs. We identified 19 signaling pathways that were enriched in moDCs of SLE. The top five pathways were PI3K-Akt signaling pathway, cytokine-cytokine receptor interaction, protein digestion and absorption, extracellular matrix-receptor interaction, and hematopoietic cell lineage (Fig. 3d).

### Construction of the coexpression network with differentially expressed lncRNAs and mRNAs

Next, we constructed a coexpression network of these coding-noncoding genes that included the differentially expressed lncRNAs and mRNAs. Our data showed that the coexpression network was composed of 127 network nodes and 316 connections between 62 lncRNAs and 65 mRNAs. This coexpression network indicated that one lncRNA could target, at most, 16 coding genes and that one coding gene could correlate with at most 12 lncRNAs (Additional file 3: Figure S1). NR_024243 and lnc-BSPH1–2:1 were the most connected lncRNAs. NMT1, NPM3, and UPB1 were the most connected mRNAs.

### Analysis of lncRNA-target gene regulatory network

We identified the chromosomal coexpression genes 10 kbp upstream and downstream of the differentially expressed lncRNAs to determine potential lncRNA “*cis*” genes. The results of the “*cis*” analyses are shown in Additional file 4 (Table S3). Then we predicted the *trans*-associated genes of the differentially expressed lncRNAs with RNAplex v0.2, and the results are shown in Additional file 5 (Table S4). Matured moDCs could secrete an abundant source of both inflammatory and lymphoid chemokines, sustaining interaction of naive and activated T cells with antigen-presenting matured moDCs. We then constructed lncRNA-target genes of cytokines and chemokines using Cytoscape software (Additional file 6: Figure S2). Our data show that the lncRNA-target gene network was composed of 83 network nodes and 318 connections between 55 lncRNAs and 28 mRNAs. Within this coexpression network, CXCL10 was the most chemokine connected by lncRNAs and genes and IL-6 was the most cytokine connected by lncRNAs and genes. Lnc-BSPH1–1:1, NR_024214, and ENST00000501122.2 were the most connected lncRNAs.

### Confirmation of differentially expressed lncRNAs by qRT-PCR

**Fig. 4:**
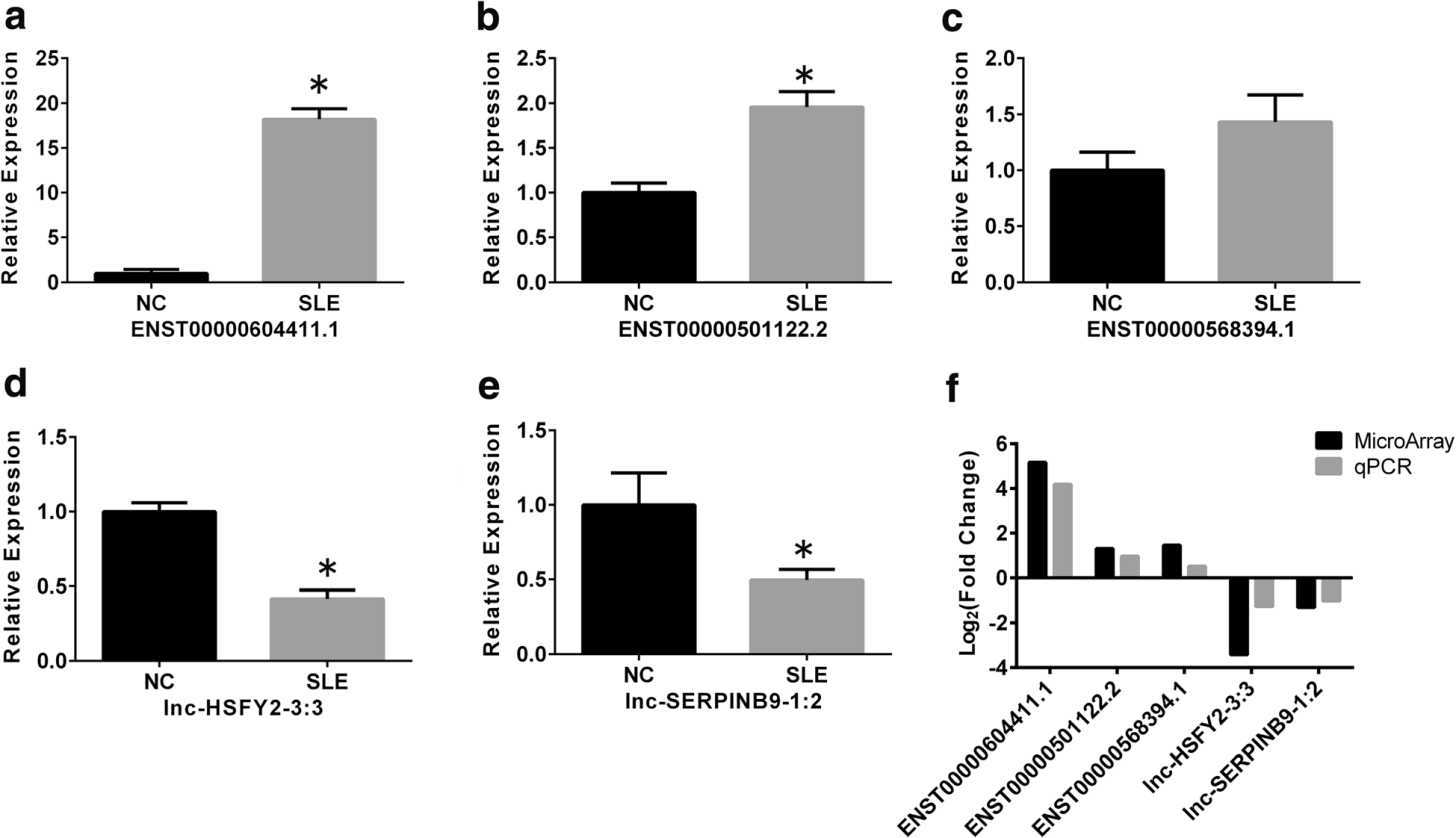
Validation of selected lncRNAs by qRT-PCR. The levels of ENST00000604411.1 (*P < 0.001), ENST00000501122.2 (*P < 0.001), ENST00000568394.1 (*P* = 0.151), lnc-HSFY2–3:3 (*P < 0.001), and lnc-SERPINB9–1:2 (*P = 0.040) were determined in moDCs of 15 normal controls (NC) and 15 patients with systemic lupus erythematosus (SLE) (**a**–**e**). Data are shown as mean ± SD. **f** The change patterns between microarray analysis and quantitative polymerase chain reaction (qPCR)

To confirm the reliability of the microarray data and considering the fold changes and the potential target genes of lncRNAs, we enlarged the sample sizes and selected five differentially expressed lncRNAs (three upregulated and two downregulated) and measured their expression level using qRT-PCR among 15 patients and 15 healthy controls including those 5 patients and 5 normal controls in the microarray. Consistent with the microarray data, the expression level of ENST00000604411.1 and ENST00000501122.2 were upregulated, and the expression level of lnc-HSFY2–3:3 and lnc-SERPINB9–1:2 were downregulated. However, the expression level of ENST00000568394.1 showed the same changed pattern as seen in the microarray analysis with no statistical significance (Fig. 4).

### Correlation between the aberrantly expressed lncRNAs and SLEDAI score of patients with SLE

**Fig. 5:**
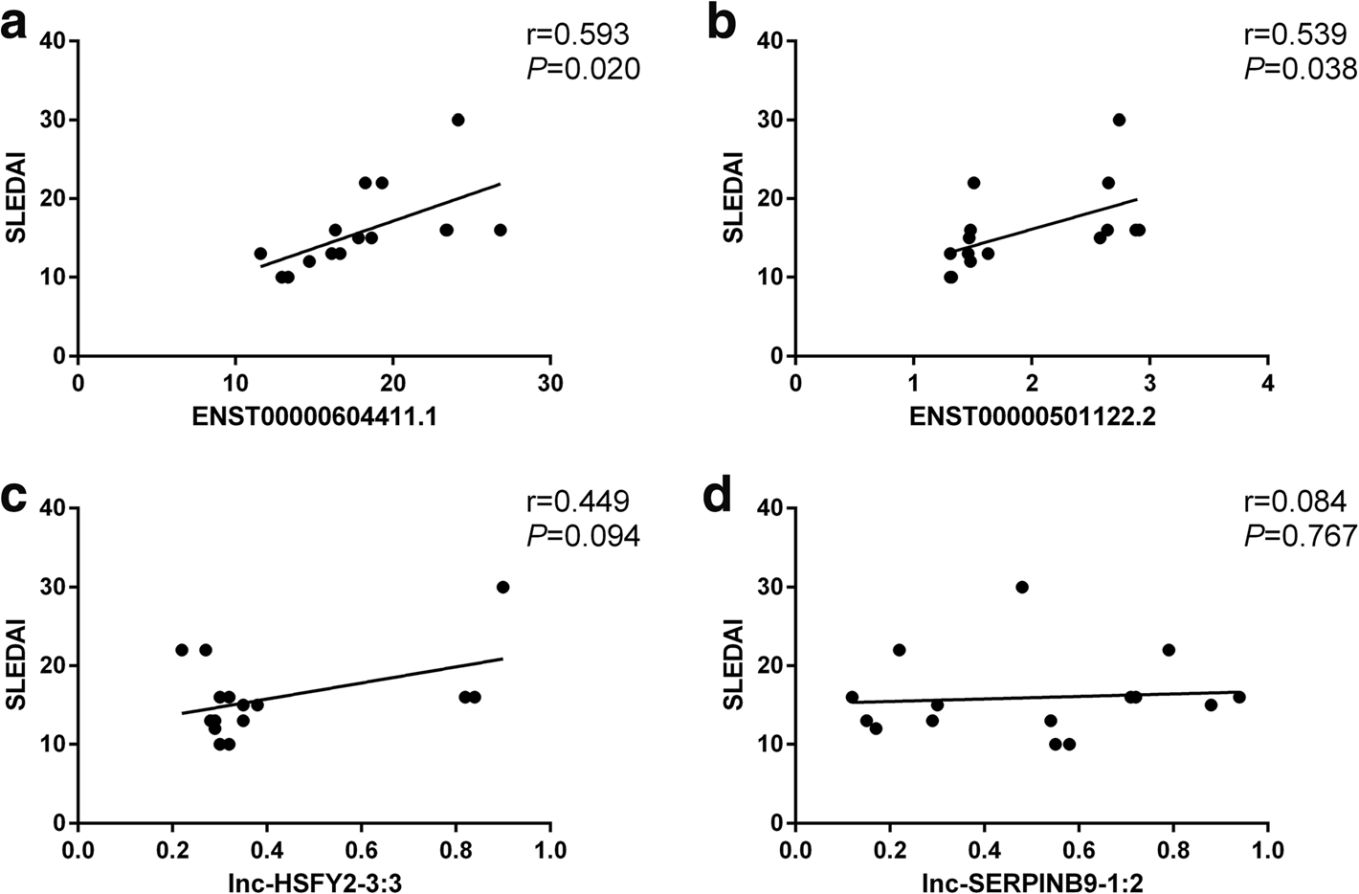
Correlation between lncRNAs and systemic lupus erythematosus disease activity index (SLEDAI). **a**, **b** ENST00000604411.1 and ENST00000501122.2 expression was positively correlated with SLEDAI score. **c** No significantly correlation was observed between lnc-HSFY2–3:3 and SLEDAI score. **d** No significantly correlation was observed between lnc-SERPINB9–1:2 and SLEDAI score

We further analyzed the correlation between the expression levels of the lncRNAs ENST00000604411.1, ENST00000501122.2, lnc-HSFY2–3:3, lnc-SERPINB9–1:2 and SLEDAI score. The expression levels of ENST00000604411.1 (*r* = 0.593, *P* = 0.020) and ENST00000501122.2 (*r* = 0.539, *P* = 0.038) were positively correlated with the SLEDAI score. However, neither lnc-HSFY2–3:3 nor lnc-SERPINB9–1:2 was significantly correlated with SLEDAI score (Fig. 5).

## Discussion

Long noncoding RNAs, more than 200 nucleotides in length, are nonprotein-coding transcripts with a lack of an open reading frame. They can regulate gene expression at the level of chromatin remodeling, gene transcription, protein transport, and trafficking [14]. There is increasing interest in the potential involvement of lncRNAs in a number of complex human diseases, including autoimmune diseases, neurological disorders, coronary artery disease, and various cancers [11, 14, 15]. Genetic evidence suggests that lncRNA GAS5, a prime candidate for the chromosome 1q25 SLE locus, is related to susceptibility for SLE [16]. GAS5 also has been linked with an increased susceptibility to SLE in mouse models, presumably as a result of its effect on the immunosuppressant role of glucocorticoids [17]. The increased lncRNA NEAT1 expression in monocytes is related to the elevated production of a number of cytokines and chemokines in SLE patients [18]. LncRNA MALAT-1 expression was abnormally increased in monocytes of SLE patients, and silencing MALAT-1 significantly reduced the expression of IL-21 in primary monocytes of SLE patients by regulating SIRT1 signaling [19]. This all suggests that lncRNAs could contribute to the pathogenesis of SLE. Furthermore, lncRNAs could also serve as potential biomarkers in SLE. For instance, Linc0949 is decreased in PBMCs of patients with SLE. It can significantly increase following effective treatment for lupus, suggesting its potential as a biomarker for diagnosis, disease activity, and therapeutic response in SLE [20]. GAS5, linc0597, and lnc-DC in plasma may also specifically identify patients with SLE [21].

Published studies involving aberrantly expressed lncRNAs in SLE patients have mainly focused on PBMCs [20, 22], T cells [23], monocytes [18], and plasma [21]. However, there are no current studies regarding lncRNAs of DCs in SLE patients. Since lncRNAs appear to be expressed in a much more cell type-specific manner than transcription factors and other protein-coding genes, the aim of our study was to explore aberrant lncRNA expression in moDCs of SLE patients to provide new insight into the pathogenesis of SLE.

We analyzed five moDC samples from individual SLE patients and five moDC samples from normal controls using lncRNA and mRNA microarrays. Based on the microarray data, we found 163 lncRNAs and 137 mRNAs that were differentially expressed. GO and KEGG pathway analyses showed that the differentially expressed mRNAs on moDCs mainly related to T-cell costimulation, chemokine activity, and cytokine-cytokine receptor interaction that are clearly associated with SLE pathogenesis.

We used qRT-PCR to validate the lncRNA microarray results in 15 patients and 15 normal controls, including those in the microarrays. Based on the qRT-PCR results, ENST00000604411.1, ENST00000501122.2, lnc-HSFY2–3:3, and lnc-SERPINB9–1:2 were differentially expressed, which was in agreement with the microarray results. The expression level of ENST00000568394.1 showed the same change pattern as shown in the microarray analysis with no statistical significance, which is likely due to the fact that the expanded test sample size for the qRT-PCR might have excluded some of the false positive results obtained in the microarray or due to technical limitations, such as cross-hybridization, signal saturation, and limited dynamic range in the microarray.

ENST00000604411.1, known as TSIX or LINC00013, expresses a noncoding antisense transcript across the 3′ end of the XIST locus. TSIX was overexpressed in systemic sclerosis (SSc) dermal fibroblasts both in vivo and in vitro, and is higher in SSc sera. TSIX is a new regulator of collagen expression which stabilizes the collagen mRNA [24]. It also protects the active-X from ectopic silencing once X-inactivation has commenced [25]. There is an increased incidence and prevalence of systemic lupus erythematosus in females, which might involve X chromosome inactivation [26]. In our study, we found ENST00000604411.1 was increased in moDCs of SLE patients and the expression level of ENST00000604411.1 was positively correlated with the SLEDAI score. Therefore, the upregulated ENST00000604411.1 might facilitate X chromosome inactivation through protecting the active-X from ectopic silencing and take part in the pathogenesis of SLE; however, further studies need to be performed to know exactly what role TSIX plays in the processes of the disease.

ENST00000501122.2 is a 22.74-kb intergenic lncRNA transcript, known as NEAT1. This lncRNA is retained in the nucleus where it forms the core structural component of the paraspeckle suborganelles. Zhang et al. [27] found NEAT1 expression was abnormally increased in SLE patients and predominantly expressed in human monocytes. There was also a positive correlation between NEAT1 and clinical disease activity in SLE patients. Furthermore, silencing NEAT1 significantly reduced the expression of a group of chemokines and cytokines, including IL-6, CXCL10, etc., which were induced by LPS continuously and in late stages [27]. NEAT1 is also critical for the expression of IL-8 [28]. In our study, we found NEAT1 expression was increased in moDCs from SLE patients. Therefore, NEAT1 was upregulated in both moDCs and their parent monocytes in SLE. We have also previously found IL-6 expression was increased in moDCs of SLE patients [8]. Future studies should focus on whether the increased NEAT1 expression in moDCs impact the cells to produce increased cytokines and chemokines in SLE patients.

Widespread change in lncRNAs might regulate the gene expression and production of inflammatory mediators in moDCs. Lnc-DC knockdown impacted the antigen uptake function of moDCs, impaired allogenic CD4^+^ T-cell proliferation, and reduced the strength of cytokine release [12]. A previous study demonstrated that lincRNA-Cox2, a critical inflammation mediator, was induced in bone marrow-derived DCs after stimulation with LPS [29]. A further study revealed that lincRNA-Cox2 mediated both the activation and repression of distinct classes of immune genes, including Irf7, CCL5, and IL-6, etc. [30]. Moreover, lncRNAs, such as THRIL, PACER, and lincRNA-EPS, can also regulate the inducible expression of cytokines following immune activation [31]. In the current study, we found that the predicted target genes of differentially expressed lncRNAs in moDCs included cytokines and chemokines, especially IL-6, CXCL10, IL-10, CXCL2, etc. We also observed that the differentially expressed mRNAs in moDCs of SLE patients were enriched in the process of cell migration and chemokine activity and in the pathways of cytokine-cytokine receptor interaction. This is similar to our previous observation that moDCs in SLE manifested proinflammatory functions such as producing elevated levels of IL-6, CCL2, and CCL5, with the attraction of more CD4^+^ T cells compared with moDCs of healthy controls [8]. In addition, the majority of patients with SLE display an increased expression of type I interferon (IFN)-regulated genes, also known as an IFN signature [32]. We found that some target genes of the differentially expressed lncRNAs in moDCs are connected to the type I IFN system, such as IRF5 and TREX1. IRF5, the target gene of lncRNA NR_034053.2, is associated with increased serum IFN activity in SLE patients [33]. The target gene of lncRNA n339353 is TREX1, and loss-of-function mutations in this leads to accumulation of intracellular DNA that triggers type I IFN production [34]. The expression level of both lncRNA NR_034053.2 and lncRNA n339353 is elevated in moDCs of SLE patients, which might suggest a regulatory role of moDCs in producing type I IFN in SLE. Since cytokine production is one of the major functions of DCs with great biological importance, more studies are needed to focus on the crosstalk between cytokines and lncRNAs in DCs; the functions of these differentially expressed lncRNAs require further study.

The limitation of our study is that we have not examined lncRNA profiles in circulating myeloid DCs (mDC) and plasmacytoid DCs (pDC) in SLE patients, which is more meaningful in the clinic. However, since the population of mDC and pDC is very small in the peripheral blood, it is difficult to obtain enough cells for study. We therefore utilized the well-accepted model of human DC differentiation from peripheral blood monocytes under inflammatory conditions [12], and we have successfully identified those cells as DCs according to their morphology, phenotype, and function. The potential disadvantage of using moDCs is that it is an artificial system and could be affected by external environments. However, moDCs in our study were differentiated under the same conditions. As a result, the discrepancy between the SLE group and the healthy control group might be due to the intrinsic factors. Another major limitation is that all SLE patients and healthy controls in our study are Chinese females, and so there is gender bias in our findings. Our results may not reflect to male patients and patients of other ethnic backgrounds. Since DCs are the master regulators for initiation, amplification, and perpetuation of SLE [35], targeting DCs may be of benefit for treatment in the future.

## Conclusions

Our study provides comprehensive lncRNA and mRNA profiles for moDCs in SLE patients. The differential expression of ENST00000604411.1, ENST00000501122.2, lnc-HSFY2–3:3, and lnc-SERPINB9–1:2 may participate in the pathogenesis of SLE. The expression level of ENST00000604411.1 and ENST00000501122.2 was positively correlated with SLEDAI score and may have disease activity evaluation value for SLE.

## Additional files

Additional file 1: **Table S1.** The specific information about the differentially expressed lncRNAs. (XLSX 76 kb)

Additional file 2: **Table S2.** The specific information about the differentially expressed mRNAs. (XLSX 83 kb)

Additional file 3: **Figure S1.** Coexpression network with differentially expressed lncRNAs and mRNAs. Red triangles represent upregulated lncRNA, green triangles represent downregulated lncRNA, circles with a yellow border represent upregulated mRNA, and circles with a blue border represent downregulated mRNA. Arrows represent positive correlation and terminated lines represent negative correlation. (PDF 33 kb)

Additional file 4: **Table S3.** The predicted cis-associated genes of the differentially expressed lncRNAs. (XLSX 2714 kb)

Additional file 5: **Table S4.** The predicted trans-associated genes of the differentially expressed lncRNAs. (XLSX 7838 kb)

Additional file 6: **Figure S2.** LncRNAs and targeted genes regulating cytokine and chemokine networks. The node color differs from dark green to dark red according to the connection numbers from small to large. Squares represent target genes. Circles represent lncRNAs. Arrows represent positive correlation and terminated lines represent negative correlation. (PDF 32 kb)

## Abbreviations

APC: Antigen-presenting cell
DC: Dendritic cell
FDR: False discovery rate
GAPDH: Glyceraldehyde 3-phosphate dehydrogenase
GO: Gene ontology
IFN: Interferon
IL: Interleukin
KEGG: Kyoto Encyclopedia of Genes and Genomes
lncRNA: Long noncoding RNA
LPS: Lipopolysaccharide
moDC: Monocyte-derived dendritic cell
PBMC: Peripheral blood mononuclear cell
qRT-PCR: Quantitative real-time polymerase chain reaction
SLE: Systemic lupus erythematosus
SLEDAI: Systemic lupus erythematosus disease activity index
SSc: Systemic sclerosis
