# Supplementary figures and images for: Long noncoding RNA expression profile and association with SLEDAI score in monocyte-derived dendritic cells from patients with systematic lupus erythematosus
Source: Arthritis Res Ther. 2018 Jul 11;20:138. doi: 10.1186/s13075-018-1640-x (PMC6042324; doi:10.1186/s13075-018-1640-x)

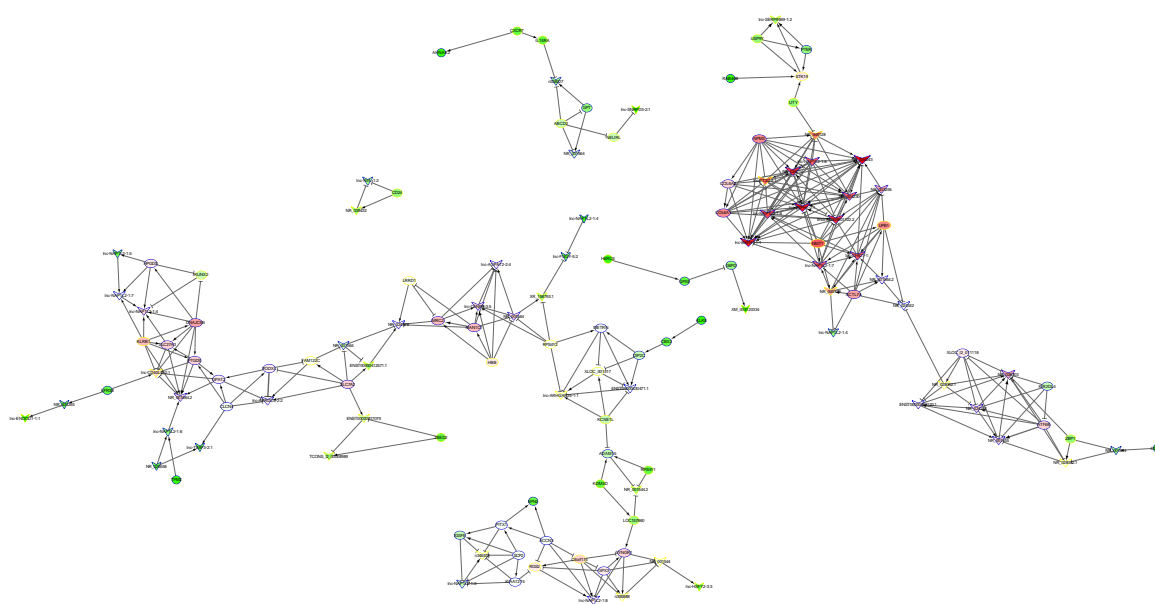

Supplement: Supplementary file 3 — Figure S1. Coexpression network with differentially expressed lncRNAs and mRNAs. Red triangles represent upregulated lncRNA, green triangles represent downregulated lncRNA, circles with a yellow border represent upregulated mRNA, and circles with a blue border represent downregulated mRNA. Arrows represent positive correlation and terminated lines represent negative correlation. (PDF 33 kb) [file 13075_2018_1640_MOESM3_ESM.pdf]

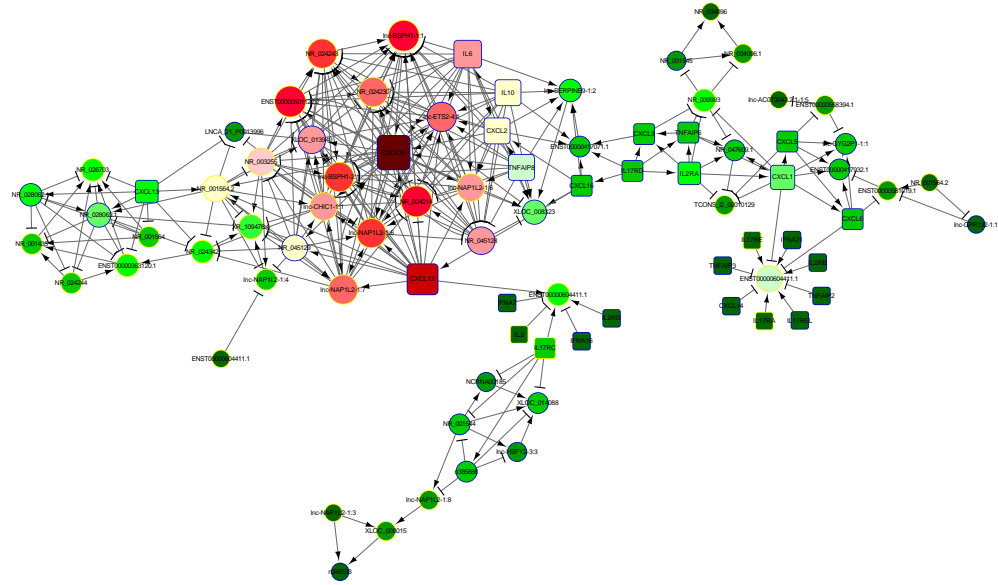

Supplement: Supplementary file 6 — Figure S2. LncRNAs and targeted genes regulating cytokine and chemokine networks. The node color differs from dark green to dark red according to the connection numbers from small to large. Squares represent target genes. Circles represent lncRNAs. Arrows represent positive correlation and terminated lines represent negative correlation. (PDF 32 kb) [file 13075_2018_1640_MOESM6_ESM.pdf]
